# Classification of Textile Polymer Composites: Recent Trends and Challenges

**DOI:** 10.3390/polym13162592

**Published:** 2021-08-04

**Authors:** Nesrine Amor, Muhammad Tayyab Noman, Michal Petru

**Affiliations:** Department of Machinery Construction, Institute for Nanomaterials, Advanced Technologies and Innovation (CXI), Technical University of Liberec, 461 17 Liberec, Czech Republic; nesrine.amor@tul.cz (N.A.); michal.petru@tul.cz (M.P.)

**Keywords:** classification, fiber reinforced polymer composites, artificial neural network, fuzzy logic, Sequential Monte Carlo methods

## Abstract

Polymer based textile composites have gained much attention in recent years and gradually transformed the growth of industries especially automobiles, construction, aerospace and composites. The inclusion of natural polymeric fibres as reinforcement in carbon fibre reinforced composites manufacturing delineates an economic way, enhances their surface, structural and mechanical properties by providing better bonding conditions. Almost all textile-based products are associated with quality, price and consumer’s satisfaction. Therefore, classification of textiles products and fibre reinforced polymer composites is a challenging task. This paper focuses on the classification of various problems in textile processes and fibre reinforced polymer composites by artificial neural networks, genetic algorithm and fuzzy logic. Moreover, their limitations associated with state-of-the-art processes and some relatively new and sequential classification methods are also proposed and discussed in detail in this paper.

## 1. Introduction

Classification of textiles and polymer based nanocomposites by computer added programs is relatively a new approach that develops the simulations of human brain in the form of algorithms to solve complex problems. Machine learning is a subcategory of artificial intelligence that provides the solution of various issues i.e., grading, classification, defects detection, quality control, prediction and process optimization, through advanced tools such as image processing, soft computing and computer vision algorithms. The adaptation of machine learning in textiles has aroused in recent years [1]. Durable, sustainable and quality products are produced with the help of machine learning algorithms with minimal effort. These algorithms are essential parts of modern artificial intelligence systems and researchers have been significantly used these systems for the betterment of textiles. Therefore, machine learning based automatic fabric defects detection system are integrated in modern textile machines to evaluate fiber grading, yarn quality and fabric performance.

Due to their economic benefits, textiles and polymers based composites have received tremendous attention and researchers used them in various fields due to their excellent mechanical, electrical and interfacial properties [2,3,4,5,6]. In terms of current research, the interfacial performance of fibre/resin for composites was observed to be sensitive to the actual service environment. The potential fibre/resin debonding may occur. In addition, the fatigue resistance is a key advantage of textile-based composites compared to the steel materials, that expands the application circle of composites in automobiles, aerospace, oil extraction industry, civil and building industry [7]. A group of researchers worked with the interfacial, mechanical and thermal properties of fibre reinforced composites and reported interesting results [8,9]. Li et al. worked with interfacial shear strength of pultruded rod made of carbon/glass. They investigated the effect of hydraulic pressure and water immersion on interfacial properties. The results revealed that the hydraulic pressure had positive impact on the interfacial performance of the carbon/glass composite and conversely, water immersion reduced the interfacial strength of carbon/glass rod [10]. In a recent study, Li et al. expanded their work on carbon/glass pultruded rod and investigated the interfacial, thermal and mechanical properties at elevated temperature. They reported that at elevated temperature, interfacial shear strength decreased with time for hybrid fibre reinforced composites. Longer exposure led to more degradation and plasticizing as well as hydrolysis observed due to the diffusion of water molecules [11]. In another study, Li et al. worked with the mechanical properties and life service evolution of unidirectional hybrid carbon/glass pultruded rod under harsh and elevated conditions. The results showed that fibre/resin debonding occurred under longer exposure and overall mechanical, thermal and interfacial properties decreased [12].

**Figure 1:**
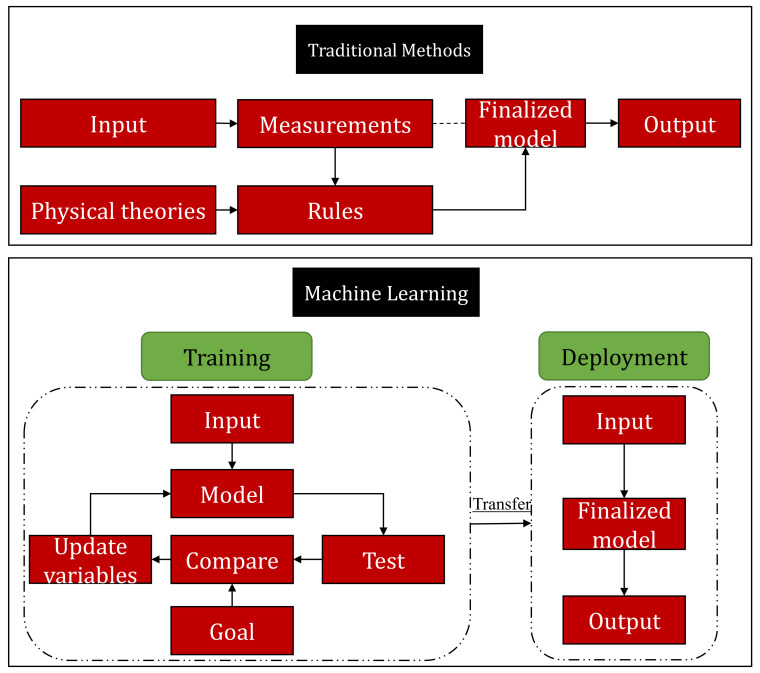
Comparison of machine learning approaches with traditional deterministic models.

The use of machine learning in textiles, especially for classification, has shown its potential exponentially in the current era [13,14]. Zimmerling et al. reported the application of Gaussian regression algorithm to improve the geometrical shapes of fiber reinforced textile composites. This method significantly improved the assessment criterion for fibre reinforced plastics components. Based on the efficiency, they suggested machine learning as an economical tool than finite element method, for the evaluation of textile processes [15]. In another work, Seçkin et al. reported a production fault in gloves industry. They used time-series data for the simulation and forecasting of this problem and classified it with different machine learning algorithms [16]. Ribeiro et al. proposed an automatic method to predict different properties of woven fabrics based on design and finishing features [17]. Due to the complexities of their micro-structures and boundary conditions, the classification of overall characteristics of textiles and polymer composites is still a challenging task even for machine learning. Therefore, a highly efficient and accurate approach is required that can predict the microscopic structural performance under different geometries. Apart from the above discussion, hardware utilization and technical issues are two other major constrains during the application of machine learning in textiles. However, to address these problems, various machine learning and computer vision-based applications are reported in the literature as deterministic and non-deterministic models. Mathematical models, empirical models and computer aided models i.e., finite element method (FEM), are deterministic models. However, genetic algorithm (GA), artificial neural network (ANN), chaos theory (CT) and fuzzy logic (FL) are non-deterministic approaches. Figure 1 shows the difference between machine learning approaches and traditional deterministic engineering models.

In recent years, considerable research efforts have been made to the development of machine learning tools for classification, prediction and defects detection. Although, the prediction and defects detection for textiles and polymer composites have been reviewed, the comprehensive review on the classification of fiber reinforced polymer composites is still missing. Therefore, our motivation is to provide a detail description about the used algorithms for fiber reinforced composites. This may help our readers to find out best possible algorithm for their future research endeavours. We categorized the applications of machine learning and computer vision algorithms into four classes based on standard textile manufacturing processes i.e., spinning, weaving, finishing and fiber reinforced polymer composites. In each of these four classes, we elucidated the recently reported work for defects detection, identification, classification and prediction by image segmentation-based approaches, color-based approaches, texture-based defect detection and by deep learning. In addition, we reviewed the limitations of existing state of the art methods and proposed a possible future research direction in textile composites using the sequential Monte Carlo methods.

**Figure 2:**
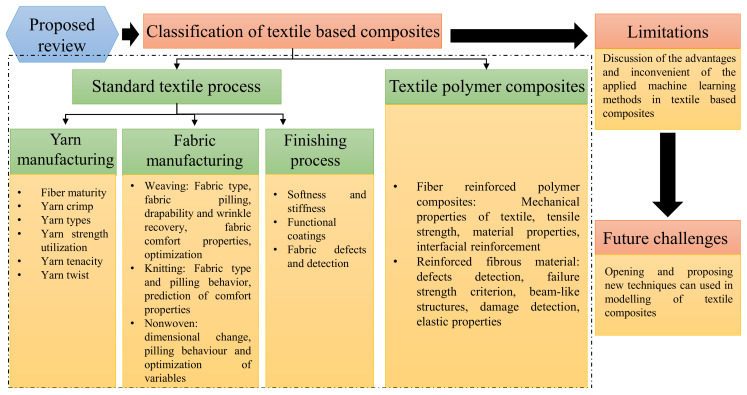
A schematic illustration of this study.

After the introduction, this paper is organized in a following sequence: Section 2 focuses the utilization of machine learning in spinning, weaving, non-woven and textile finishing applications. The following Section 3 provides the limitations of widely used approaches. Section 4 explains the possibilities of future challenges for sequential Monte Carlo methods in textiles and polymer-based composites and the final Section 5 summarizes the paper with few suggestions to overcome the future challenges. Figure 2 explains the scheme that elucidates the methodology of the proposed paper. The authors believe that the approach delineates here opens up a new gateway for researchers to choose the best suitable machine learning tool in order to work with textile substrates and composites.

## 2. Classification Based on Textile Processes

The necessity to process raw data and explore valuable information from it has become essential in every field of science, engineering, business and medicine. In textiles, even when a simple product e.g., a t-shirt is considered, bulk amount of data is generated from raw materials, quality parameters and machine settings. The data could be nonlinear and multivariable depend on the relationship between fiber properties and yarn properties or between fabric performance and machine settings. Moreover, improvements and innovations in textiles with the introduction of technical textiles have occurred with exceptional performance expectations at extreme conditions e.g., against sun, cold, impact, knife, bullet and microorganisms [18,19]. Therefore, the demand for data processing and discovery of valuable information from this data is continuous in textile industry. Many traditional models e.g., statistical and mathematical, have been reported in numerous studies to process textile data. However, these classical models remain incapable of discovering the complex relationship between the variables. To solve this challenge, machine learning models are implemented in almost all areas of textile engineering. In textile processes, the trend of reported literature elucidates that the researchers tried to establish a critical relationships among essential parameters of fibers, yarns and fabrics. Machine learning algorithms are robust and powerful tools for modeling and solving complex and nonlinear applications. In this context, Majumdar published a book on soft computing in textile engineering [20]. This book compiled various research studies based on ANN and FL approaches during yarn modelling, fabric manufacturing, garments modelling by FL, composites modelling for quasi-static mechanical properties, viscoelastic behaviour and fatigue behaviour using ANN, textile quality evaluation using image processing, ANN and FL approaches.

ANN has been widely studied for textile data since last decade and helped the researcher to get better efficiency for fiber classification, defect detection, prediction and modeling of yarn, fabric, color matching, color separation and their coordinates conversion [21]. Vassiliadis et al. introduced a comprehensive overview of ANN applications in fabric manufacturing [22]. ANN had been successfully utilised for fibrous properties (classification of fibers, color grading, selection of cotton bales, identification of control parameter), yarns parameters (detection of faults, prediction of tensile properties and shrinkage) and for fabrics properties (defects detection, prediction of thermophysiological, sensorial and comfort properties, bursting of woven and knitted fabrics).

The literature discussed above provides an overview of machine learning till 2011. Therefore, we will present a review of recent advanced works in this field. In this section, we categorized the textile applications using machine learning algorithms into four classes based on standard textile-based processes i.e., spinning, weaving, finishing and fiber reinforced polymer composites.

### 2.1. Classification Based on Yarn’s Production

Yarn is generally considered as a primary element for the manufacturing of high quality textiles and in recent years, numerous studies were conducted on the classification, modeling and prediction of essential yarn parameters as given below:

#### 2.1.1. Fibre Maturity

Fibre maturity is an important and significantly crucial parameter especially when the researchers deal with yarns properties and desire excellent final product. In general, fibre maturity is considered as a functional and primary building block of any good textile product. Therefore, many researcher worked with the prediction of fibre maturity. Farook et al. proposed that ANN algorithms are excellent prediction tools in order to predict cotton fibre maturity [23]. They selected various fibre characteristics as an input variables and analysed fibre maturity as an output variable. The simulation results showed that ANN predicted cotton fibre maturity was higher when compared with the experimental values. However, there was no optimal result in this application.

#### 2.1.2. Yarn Crimp

Malek et al. evaluated the performance of ANN for the prediction of yarn crimp in woven barrier fabrics [24]. They performed two experiments to predict yarn crimp. The purpose of both experiments was to predict the crimp in warp yarn and weft yarn respectively. The input variables were weave style, density of warp and weft yarns, fibre and filament fineness, shed time and loom speed. However, the only exception in the second experiment was the replacement of yarn fineness with filament fineness. ANN results showed good results for the prediction of yarn crimp with the exception of two small deviations between actual and predicted output. In a different work, Majumdar et al. introduced mathematical, statistical and ANNs models to predict breaking force at elongation of ring-spun cotton yarns [25]. The inputs for these three models were yarn count and cotton fiber properties. ANN model provided a better prediction performance compared to the statistical and mathematical models. Later, they reported the implementation of a hybrid neuro-fuzzy system for the prediction of yarn strength [26]. The results were compared with standard ANN and regression models for prediction accuracy. ANN showed better prediction results than others.

#### 2.1.3. Yarn Types

Before the production of yarn, the prediction of quality parameters is important to overcome production faults. In an experimental study, Almetwally et al. used ANN and linear regression for the prediction of core spun yarn strength, elongation and rupture [27]. The results showed that ANN models provided significantly accurate prediction for yarn strength. Recently, Doran et al. reported the utilization of ANN and support vector machine (SVM) methods to avoid faulty fabric production [28]. In addition, they used statistical tools i.e., analysis of variance (ANOVA) and principal component analysis (PCA) to overcome input dimensions. The test results showed that both ANN and SVM methods provided effective predictions for yarn quality characteristics. However, SVM showed slightly better results than ANN for mean absolute percentage error (MAPE) and coefficient of correlation (R).

#### 2.1.4. Yarn Tenacity

Dashti et al. worked with yarn tenacity through ANN and produced a decision support system by applying GA [29]. Experimental results showed that ANN offered an accurate prediction for yarn tenacity with less than 3.5% error. In addition, GA was applied to obtain optimal input parameters for yarn production. The obtained tenacity was greater than the desired tenacity, therefore, a reduction in production cost was observed. The implementation of this strategy was useful to find good input conditions in order to achieve desired tenacity.

#### 2.1.5. Yarn Strength Utilization

Mishra used ANN models during the production of cotton fabric for the prediction of yarn strength utilization [30]. The selected input parameters were yarn counts, initial crimps, total number of yarns and yarn strengths in longitudinal and transverse directions along with the weave float length. The experimental results showed that yarn strength utilization percentage increased with an increase in yarn number in both directions. However, a decrease in crimp percentage and float length was observed. Mozafary et al. proposed a combined approach, where they used K-means algorithm for data clustering and ANNs for defects detection i.e., yarn unevenness [31]. The feedforward ANN and Levenberg–Marquardt training function of back propagation were applied in this method and the effectiveness was demonstrated by a comparative analysis with standard ANN results. In an experimental study, Malik et al. applied a back propagation ANN to analyse the prediction efficiency of used model for tensile properties of even and uneven yarns extracted from polyester-cotton blend [32]. The selected parameters were twist multiplier, cot hardness and Break draft ratio. The reported results of linear regression and ANN for tensile properties were compared with standard methods. El-Geiheini et al. worked with different types of yarns and used ANN and image processing tools for modeling and simulation of yarn tenacity and elongation [33]. They reported that the proposed techniques were suitable for the estimation of various yarn properties with minimum error. In another study, Erbil et al. used ANN and regression tools for tensile strength prediction of ternary blended open-end rotor yarns [34]. They applied multiple linear regression (MLR) and trained ANN algorithm with Levenberg–Marquardt backpropagation function. Furthermore, they compared both models for prediction efficiency. The results demonstrated that ANN models gave better prediction output than MLR for both parameters i.e., breaking strength and elongation at break.

#### 2.1.6. Yarn Twist

Yarn twist i.e., S twist, Z twist etc., is another important and noteworthy parameter in order to estimate end product’s performance. Therefore, different researcher worked with this variable and reported interesting results. Azimi et al. used ANN in order to predict twist type for textured yarns [35]. They investigated the effects of heater temperature, texturing speed and the effects of twist type on yarns crimp stability for hybrid yarns. The testing results demonstrated that ANN models were excellent for the prediction of yarn properties under selected variables.

### 2.2. Classification Based on Fabric Manufacturing

In this part, we classified the use of machine learning tools into three categories based on fabric manufacturing methods i.e., weaving, knitted and non-woven.

#### 2.2.1. Weaving

The process of weaving is based on interlacing of yarns in warp and weft directions. However, with time, textile woven structures have become more and more complex by the addition of diagonal yarns in interlacing. Therefore, prediction of woven textiles is now a complex task that requires accumulated empirical knowledge about various parameters of woven textiles. Some of those variables are listed below where researchers performed machine learning algorithms to gain better performance and utilization of woven textiles.

##### Fabric Type

Woven structures are the mostly used structures not only in textile production but also in composites [36,37,38,39,40,41]. Ribeiro et al. proposed an automated machine learning method to predict the physical properties of woven fabrics based on finishing features and textile design. They investigated nine different properties including pilling, abrasion and elasticity and reported improved prediction results for all properties with low prediction error. They applied Cross-Industry Standard Process for Data Mining (CRISP-DM) iterations, where every iteration was based on the verification of standard input parameters. At CRISP-DM stage, an automated machine learning (AutoML) algorithm was performed to choose optimal regression model among six different machine learning algorithms. The results demonstrated that significantly better output was achieved by the selected codes for fixed sequence of yarns and fabric finishing treatment [17]. In an experimental study, Hussain et al. proposed a novel machine learning algorithm depends on transfer learning and data augmentation in order to recognize and classify the complex patterns of woven textiles. The texture and pattern of textiles are considered as essential factors to design and produce high-quality fabrics. The proposed algorithm worked with residual network through which textures of woven fabric were extracted and auto classified as an end-to-end manner. They suggested that the reported results would be effective even all of the fabric properties are altered [42].

##### Fabric Pilling, Drapability and Wrinkle Recovery

Fabric pilling, drapability and wrinkle recovery are the aesthetic properties of textiles and considered as performance indicator of textile fabrics for quality evaluation. Eldessouki et al. applied adaptive neuro-fuzzy models (ANFIS) for the evaluation of pilling resistance of woven fabrics. In this proposed approach, they classified the selected samples on the basis of texture patterns and compared their results with standard method of pilling resistance for correlation [43]. Xiao et al. predicted cotton-polyester fabric pilling with ANN (Back-propagation approach) and then used GA for optimization of their results. The optimized results revealed that GA algorithms were better in terms of root mean square error (RMSE), MAPE and mean absolute error (MAE) compared to ANN [44]. Drapability is one of the most important aesthetic properties that plays crucial role in providing graceful effects to textile fabrics. Drapability depends on experience and skills of humans and is judged subjectively. It renders the complexities during drape comparisons particularly when judged by different persons. Taieb et al. used ANN for the prediction of fabric drape ability under low stress. They reported that the application of ANN for the prediction of aesthetic properties including drapability is a promising one and is physical factors played crucial role during the prediction of fabric drape ability [45]. Hussain et al. compared ANN with adaptive neuro-fuzzy inference system (ANFIS) during the evaluation of fabrics wrinkle recovery [46]. They found that for both types of algorithms, the input conditions suitable for better wrinkle recovery were linear densities of both warp and weft sides. However, the suitable output variables were crease recovery angles of warp and weft yarns. The results demonstrated that simulation performed by ANN produced slightly better results than ANFIS with significant accuracy percentage. However, ANFIS process was more useful while drawing surface plots among variables. ANN algorithms do not have this feature.

##### Fabric Comfort Properties

Comfort evaluation is a noteworthy parameter in terms of fabric overall performance [47,48,49,50,51]. Majority of machine learning algorithms were applied on fabric’s thermophysiological and sensorial comfort. Malik et al. used ANN algorithm to predict woven fabrics thermophysiological property i.e., air permeability with respect to fabric construction, raw materials involved during production and process variables [52]. ANN algorithm was trained with feedforward neural function under a hybrid back propagation method composed of Bayesian regularization and Levenberg-Marquardt function. Simulation results showed that the proposed model provided promising results on test data with lower MAE. In addition, Malik et al. employed another ANN algorithm to show a relationship between loom parameters, used material and construction of fabric in terms of porosity, mean pore flow, mean pore size with air permeability [53]. The experimental result showed that ANN algorithms were excellent for the prediction of comfort properties with minimal error. Wong et al. applied a hybrid approach that combined ANN and fuzzy-logic for overall prediction of clothing comfort by considering physical properties as input variables [54]. Simulation results provided maximum correlation coefficient.

##### Optimization

Optimization of process variables in order to reduce cost and improve production efficiency is another important factor in textiles. Therefore, many studies were carried out to investigate the process of optimization. Xu et al. combined differential evolution and Kriging surrogate algorithms to study the optimization of enzyme washing and production cost incurred on indigo dyed cotton [55]. They selected Taguchi L16 orthogonal array algorithm for optimization and applied it in their study. temperature of bath and concentration of enzymes were chosen as input variables and enzymatic washing as output response. Kriging model was used to analyse the relationship between variables and results revealed that the applied method was significantly efficient for the optimization of overall cost and can be further utilized in the analysis of mean square error, absolute error and relative error.

#### 2.2.2. Knitting

Knitting is another important production method in which prediction and modelling of knitting parameters are significantly complex tasks due to diversified variables i.e., knitted structures, knitting machine variables and selected yarn attributes etc. Several researchers have used different soft computing and machine learning algorithms to predict knitted fabric’s comfort properties, spirality, pilling, bursting strength as well as other aesthetic and physical properties.

##### Fabric Type and Pilling Behaviour

Pilling is a serious fault in textile production especially in knit wear. Therefore, machine learning is a useful tool to forecast pilling behaviour of knitted fabric. Unal et al. selected single jersey knitted fabrics for the evaluation of air permeability and combined ANN algorithm with regression methods for the prediction of bursting strength of knit structures [56]. Implementation of results showed that both methods were able to predict the properties of knitted fabrics. However, ANN had a slightly positive edge when used for prediction. Yang et al. identified knitted fabric pilling behaviour by modifying ANN into deep principle components analysis-based neural networks (DPCANNs) [57]. In DPCANNs, principle components automatically tracked down the fabric initial and after pilling test properties and then neural network was applied to evaluate pilling grades. The obtained results revealed that DPCANNs had above average classification efficiency for pilling behaviour of knitted fabric. Another important work using ANN was performed by Kayseri et al. where pilling tendency was predicted by selecting fabric cover factor as an input parameter [58]. They observed that by changing cover factor, fabric pilling was controlled to a greater extent. In this study, they concluded that pilling behaviour was the outcome of pilling grade, mean pilling height as well as covered pilling area. They reported that used algorithms had very good prediction power in determining fabric pilling behaviour.

##### Prediction of Comfort Properties

Fayla et al. applied ANN algorithm on knitted fabrics to predict thermal conductivity [59]. They selected yarn conductivity, porosity, fabric weight and air permeability as input conditions. The results revealed that ANN algorithm predicted the thermal conductivity with significantly high correlation coefficient. Majumdar used ANN to predict the thermal conductivity of cotton, bamboo and their blended yarns. The input variables were bamboo fiber proportion, linear density of yarn, thickness of fabric and areal density. The correlation coefficient of this study very high [60]. Knanat et al. used ANN for the prediction of thermal resistance of wet knitted fabrics [61]. Here, they used two different ANN networks for the prediction of thermal resistance. In the first network, the input variables were moisture content, yarn, fiber and fabric parameters. However, in the second network, input variables were yarn, fiber and fabric parameters, and the output response was thermal resistance under varying moisture level. The results from both networks showed efficient prediction of thermal resistance. Mitra et al. used ANN for the prediction of thermal resistance of handloomed cotton fabric [62]. The input fabric parameters were picks per inch (PPI), ends per inch (EPI), weft and warp count. The results revealed that used ANN algorithm achieved good prediction efficiency for thermal resistance under low MAE values. In addition, EPI, warp count and weft count were major contributors for the evaluation of thermal resistance.

#### 2.2.3. Nonwoven

Machine learning algorithms have gained tremendous importance during the last few years to enhance productivity of nonwoven textiles by predicting various important parameters i.e., dimensional change, pilling behaviour and optimization of variables. Wang et al. measured the pilling of nonwoven fabrics using wavelet analysis [63]. The results demonstrated that wavelet analysis was quite similar to traditional method for pilling evaluation. Kalkanci et al. estimated fabric shrinkage by applying ANN algorithm inside relaxation methods [64]. Thermofixing, sanforizing, drying and washing were important processes applied on fabrics during finishing. Dimensional changes were predicted at the end of finishing processes by ANN. Two-layer feedforward perceptron function was used for ANN algorithm to evaluate the width of dimensional change. The experimental results showed that ANN gave better prediction results for dimensional change. Abhijit et al. applied a combination of GA and ANN as a hybrid algorithm to predict the comfort performance and the range of ultraviolet protection factor (UPF) [65]. ANN was applied as a prediction tool and GA was utilised as an optimization tool. For experimental purpose, a set of four samples were selected for the evaluation of functional properties. The proposed ANN–GA method was carried out until the required results were achieved. The results achieved by this method were in good agreement with the standard methods.

### 2.3. Classification Based on Finishing Processes

#### 2.3.1. Handle Modifications (Softness and Stiffness)

Modification of textile end product by applying softeners and stiffeners is necessary to improve the aesthetic properties. The selection of these materials attracts the scientists and researchers to build and train special machine learning algorithms, special mathematical models and soft computing tools. Farooq et al. used ANN to predict the shade change of dyed knitted fabrics after finishing application [66]. The inputs were the shade percentage, dye color and finishing concentrations. The output was delta values with respect to standard samples. Simulations results showed that ANN provided high prediction accuracy for shade change that occurred during finishing with minimal value of error between actual and predicted values.

#### 2.3.2. Functional Coatings

Malik et al. used ANN for the prediction of antimicrobial performance of chitosan/AgCl-TiO${}_{2}$ coated fabrics. The input variables were curing time and concentration of colloids [67]. Samples were developed with different blends of selected colloid under different curing time. Feedforward ANN was trained under a hybrid combination of Bayesian regularization and Levenberg Marqaurdt algorithms. The testing results had an acceptable MAE during network training. Furferi et al. introduced a novel ANN algorithm for the prediction of coating process on textile fabrics [68]. Testing results demonstrated the significance of ANN model for coating mechanism. Ni et al. proposed a novel online algorithm that detected and predicted the coating thickness of textiles by hyperspectral images [69]. The proposed algorithm was based on two different optimization modules i.e., the first module was called extreme learning machine (ELM) classifier whereas the second one was called a group of stacked autoencoders. The lateral module was designed to take data from hyperspectral images. However, ELM module optimized by a new optimizer known as grey wolf optimizer (GWO). GWO was used to determine the number of neurons and weights to get more accuracy during classification. The results explained that online detection performance significantly improved with a combination of VW-SAET with GWO-ELM that provided 95.58% efficiency.

#### 2.3.3. Fabric Defects and Detection

Fabrics are occasionally the end product of any textile manufacturing process and fabric defects inspection is very important in terms of post manufacturing processes i.e., marketing, merchandising and branding. In a simple term, Fabric defects detection is a crucial process applied to control the quality of textile production. Machine learning algorithms have also played their role in this detection/inspection process. The most famous machine learning tools used in defects detection are ANN and image processing algorithms that have been applied for defects detection and grading of woven, knitted and nonwoven textiles. Hanbay et al. presented a literature review about the methods used for the detection of fabric defects and explained that detection methods had several types including structural, hybrid, spectral, model-based and statistical [70]. Czimmermann et al. presented a detail review based on automatically detection of fabric faults and fabric defect [71]. Rasheed et al. reported a comprehensive study on faults detection methods of textiles [72]. The widely used detection methods are based on image segmentation, color coordinates, frequency domain, texture-based, image morphology operations and deep learning. Eldessouki et al. applied a defects detection method composed of a hybrid combination of sepctral (Fourier transform) and (spatial) statistical functions that detected the fabric defects from images [73]. They applied component analysis to overcome input characteristics of selected datasets. The use of PCA in this application increased the classification rate. Liu et al. proposed an algorithm composed of low-rank decomposition and multi-scale convolution neural networks for defects detection [74]. Convolution neural networks were applied to extract multiple characteristics of defects from images for the improvement of image characterization ability to deal with complex textures. However, low-rank decomposition tool was established to analyze matrix characteristics for background (low-rank part) and for (salient defects). Furthermore, the salient defects map produced by sparse matrix was further diversified under threshold to localize the defected area of fabric. The test results showed that extracted features by neural network were accurate enough to analyse fabric texture than traditional standard methods i.e., local binary pattern and histogram of the oriented gradient.

Many other researchers utilised machine learning algorithms for defects detection. Sezer et al. applied independent component analysis (ICA) for defects detection at block level using a sample image [75]. They reported that this method provided satisfactory results for plain weave fabrics. However, for twill and texture weave patterns, this method is not generalized yet. Yapi et al. proposed redundant contourlet transform (RCT) method for defects detection [76]. A finite mixture of generalized Gaussians (MoGG) was used for modeling RCT coefficients that constituted statistical signatures to differentiate the defected fabric from defect-free fabric. The proposed approach was based on three steps: (1) detection of basic pattern for image decomposition and signature calculation, (2) discrimination between defected and defect-free fabric through Bayes classifier (BC) based on labeled fabric samples, and (3) detection of defects during image inspection by testing local patches. Experimental results revealed that the used approach achieved good results compared to ICA, local binary patterns (LBPs) and slope difference distribution (SDD). Li et al. proposed Fisher criterion-based deep learning algorithm for defects detection of patterned fabrics [77]. A Fisher criterion-based stacked denoising method was used for fabric images to classify into defective and defect free categories. The experimental results showed that the accuracy of proposed method was excellent for patterned fabrics and more complex jacquard warp-knitted fabric. Han et al. proposed the stacked convolutional autoencoders for defect detection [78]. The autoencoders were trained through synthetic defected data and non-defected data by using expert-based knowledge of defect characteristics, where, input was used as a defected image produced artificially and output was the corresponding clean image. Jeffrey Kuo et al. detected the following four defects in embroidery textile patterns i.e., stitch missing, joint defect, yarn floating knit and unregistered defect recognition [79]. The results demonstrated that the applied procedure was more effective than back propagation for detects detection as it took less time to train the network. Huang et al. used machine learning tools and image analysis for pilling assessment of fleece [80]. The applied methods were discrete Fourier transform, Gaussian filtering and Daubechies wavelet, for the extraction of important features of image information i.e., pilling area, pilling density and number of pilling points. ANN and SVM were used to classify the textile grade. Experimental results showed that the use of Fourier-Gaussian method improved the efficiency of classification for ANN and SVM. Table 1 elucidates a comparison of related work for defects detection in textile processes.

### 2.4. Classification Based on Textile Polymer Composites

Composites are the most promising class of versatile and durable materials of modern age. Machine learning algorithms reduce time, cost and effort to search optimal conditions for selected variables of composite structures. Therefore, machine learning is an essential and effective tool for a comprehensive evaluation of composites. Machine learning is used to solve complex numerical and applied problems in composites. In general, the fabrication of fiber reinforced composites is considered more challenging than other anisotropic structures. Sapuan et al. presented a book on ANN applications for composite materials [88]. They reported the use of ANN for numerous tasks such as defects detection in composites and polymeric structures, localization of carbon fiber–reinforced plastics and perspex plates, prediction of mechanical behavior, aging cycles evaluation, fatigue life prediction and prediction of composites life under loading. Muzel et al. presented a comprehensive review on the applications of finite element method for composite materials, failure criteria, material properties and types of elements in aeronautics, aerospace, naval, automotive, energy, sports, civil, manufacturing and electronics [89]. Dixit et al. introduced a review on modelling approaches for the prediction of mechanical properties of textile based composites using finite element method [90]. However, there are many important parameters need to investigate for the development of new algorithms for composite materials. Therefore, In this study, we will discuss these variables in detail and propose new methods to develop machine learning algorithms for textiles and composite structures. Schimmack et al. used Extended Kalman Filter (EKF) algorithm as a virtual sensor for temperature detection, composed of metal-polymer fibre based heater structure [91]. The main purpose of this algorithm was to control temperature in case of overheating or in any other emergency condition. The results revealed the accuracy of proposed approach. In another study, Gonzalez et al. used CNN for the identification of flow disturbances of dissimilar materials in composites production [92]. Specifically, CNN was applied to detect the position, size and permeability of any embedded material on the surface of mould. In CNN, the region of dissimilar material was selected as an input variable in order to recognise disturbance flow. Altarazi et al. applied multiple algorithms at a time to predict and classify tensile strength of polymeric films of different compositions. The used algorithms were stochastic gradient descent (SGD), ANN, k-nearest neighbors (kNN), decision tree (DT), regression analysis, SVM, random forest (RF), logistic regression (LoR) and AdaBoost (AB) [93]. Experimental results demonstrated that SVM algorithm showed better prediction results. In addition, the results revealed that the classification ability of used algorithms was excellent for sorting films into conforming and non-conforming parts. Balcioglu et al. compared finite element analysis with machine learning algorithms (DT, KNN, RF, SVR) for fracture analysis of polymer composites [94]. Fracture behavior of laminated composites reinforced with pure carbon, glass and carbon/glass composition were tested and compared with standard samples. The RF algorithm showed the best result with lower MSE values compared to other algorithms.

#### 2.4.1. Fiber Reinforced Polymer Composites

The use of natural fibers as a reinforcement in polymer composites has gained commercial success in terms of durable, economical and environmentally friendly materials. Khan et al. investigated the mechanical properties of cross-ply laminated fibre-reinforced polymer composites. They developed model for the prediction of mechanical properties using ANN [95]. The composite samples were developed by altering glass fibre layers with carbon fibre layers and polyphenylene sulphide with high-density polyethylene. The fibers were used as reinforcement and polyphenylene sulphide was used as a polymer matrix. Mechanical properties i.e., hardness, flexural modulus, impact and rupture strength were investigated for both directions. The input variables for ANN model were material type, matrix layers, composition and number of reinforcement. Simulation results showed that ANN predicted the mechanical properties with low MAE. In a study, Boon et al. provided a literature review on recent advances in optimization and design of fiber-reinforced polymer composites [96]. They stated that the best approach to provide accurate results was deep learning (DL). He et al. proposed a delamination detection approach for the detection of location, size and interfacial bonding of delamination in fiber-reinforced polymer composites. This method was based on frequency changes in multiple modes [97]. They employed a combination of different algorithms i.e., support vector machine, extreme learning machine and back propagation neural network for the detection of delamination parameters. Experimental results showed that SVM algorithms provided excellent prediction and classification performance as compared to other two algorithms.

**Figure 3:**
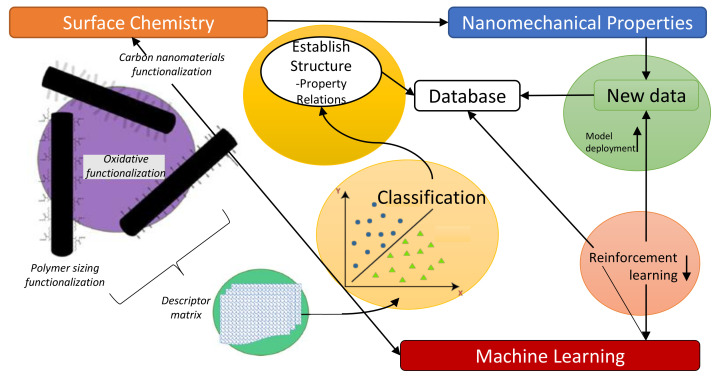
Summary of machine learning procedure validated for fiber reinforced polymer composites including CFRP, GFRP, BFRP and AFRP etc. [100].

Carbon fiber reinforced polymer composites (CFRP) are the most durable and promising modern age composite materials. By applying machine learning algorithms, researchers significantly reduced cost, efforts and time to determine optimal design points and process variables to develop CFRP structures. Mathematical modeling together with machine learning algorithms provide comprehensive analysis of CFRP structures. Matsuzaki et al. proposed an approach for state estimation and material properties of thermoset CFRP by using data assimilation [98]. Thermosetting simulation based on a non-linear state-space model that utilise ensemble Kalman filter (EnKF) for the estimation of state using data assimilation. This method estimated the degree of curing and the distribution of temperature model with thermal conductivity distribution. Simulation results showed that EnKF was successful in the estimation of the state of thermal conductivity distribution and model parameters. However, the estimation of thermal conductivity in complex distributions is still a challenging task. After the effectiveness of EnKF to estimate various CFRP thermoset molding attributes, they applied EnKF for the estimation of internal temperature during curing [99]. In this application, they selected three samples with altered thermal conductivity. The experimental results validated the efficiency of this approach using these types of specimens. Figure 3 shows a typical problem-solving method under machine learning algorithms validated for numerous types of fiber reinforced polymer composites including CFRP, glass fiber reinforced polymer composites (GFRP), basalt fiber reinforced polymer composites (BFRP) and aramid fiber reinforced polymer composites (AFRP).

Gonzalez introduced different mathematical models to detect nonlinear flexural deformation of CFRP based on stiffness level in compression and polymer matrices under different strength [101]. The study further presented modeling of different properties of fiber reinforced composite beams [102]. The proposed mathematical model described nonlinear elastic three-point bending of isotropic and reinforced beams under stiffness and strength levels. The obtained results revealed that nonlinear properties of reinforced materials and polymer matrices carefully investigated when designing real structures. Zhang et al. predicted the delaminations through Gaussian process regression (GPR) algorithm for CFRP composites during drilling [103]. Taguchi and GPR approaches explained that more data set were required for the extraction of optimal variables from fewer experimental trials. Konstantopoulos et al. used nanoindentation mapping data with machine learning algorithms to identify interfacial reinforcement [100]. Normalization and k-means clustering were applied to process data by filtering out from epoxy matrix. The used processs was trained by ANN, support vector machines and classification trees. The intrinsic modifications at the interface of CFRP proved that machine learning algorithms effectively patterned data and best fit can be obtained through SVM. Qi et al. employed the decision tree (regression tree) model to establish a relationship between variables properties and macroscopic variables of composite materials [104]. Here, representative volume element (RVE) algorithms for single-layer and multi-layer CFRP were established by a cross-scale FEM and periodic boundary conditions were loaded in order to verified the obtained results. Table 2 illustrates a comparison of related work in fiber reinforced polymer composites.

#### 2.4.2. Prediction and Estimation of Reinforced Fibrous Material

Schimmack et al. applied a prediction approach based on wavelet for defects detection of any variable in a fiber reinforced polymer composite [111]. The applied algorithm was based on variance estimation for the local Lipschitz constant of any received signal over time. In addition, a modified recursive least squares (RLS) approach was applied to evaluated the various attributes of conductive multifilament fibers used as reinforcement during production process. The results proposed that RLS algorithms were useful for the estimation of time-varying sinusoidal disturbances as well as for inductance. Lui et al. developed a new strategy to predict the initial failure strength criterion of woven fabric reinforced composites based on micromechanical model by modifying deep learning neural network (DNN) and mechanics of structure genome (MSG) [112]. MGS is used to perform initial failure analysis of a square pack microscale model that trained the samples to detect yarn failure criterion. The effectiveness of this strategy was confirmed by testing yarns of mesoscale plain weave fabrics and fiber reinforced composite materials to compute the initial failure strength constants. Soman et al. used a novel algorithm based on Kalman Filter (KF) for load estimation in beam-like structures under complex loading [113]. Simulation results using experimental data showed that the used algorithm is efficient for classification and monitoring strains in continuous welded rails. In addition, Soman et al. used Kalman filter based neutral axis (NA) tracking algorithm for damage detection in composites structures under varying axial loading [114]. The proposed scheme was applied on a composite beam instrumented with fiber optic strain sensors. The change in neutral axis location is utilized to detect delamination in beams. Simulations results showed that the proposed formulation of KF for NA tracking provided more powerful use of NA location in various applications. Hallal et al. introduced a review on analytical modeling of elastic properties of textile composites [115]. Balokas et al. proposed FEM based multiscale prediction algorithm combined with ANN for the prediction of elastic properties of textile composites under different sources of aleatory uncertainty [116]. The results of sensitivity analysis showed that the proposed algorithm provided good prediction results for elastic yarn properties. Jiang et al. proposed an approach to predict elastic modulus of fiber braided composites with uncertainties using vibration test data [117]. Reference FEM was used for simulation of uncertain elastic parameters that reflected the dynamic characteristics of a braided composites. Statistical analysis of uncertain parameters revealed that uncertainties in elastic modulus can be identified by using modal data.

## 3. Limitations of the Proposed Techniques

The textile industry is benefited from machine learning tools by using them for different applications like prediction, classification, performance simulation, structural features modelling and image analysis etc. Table 3 summarizes mostly used machine learning techniques by various researcher in textile based applications.

In general, textile processes are mostly non-linear in nature. Therefore, it is difficult to obtain analytic models for the technical design of fabrics due to the difficulties and complex structure imposed by the raw materials. Therefore, most researchers applied ANN in textiles during the confrontation nonlinear and multiparameter problems, without an analytical solution. Furthermore, the use of ANN in textile data prediction, detection, identification and classification problems covers fabrics, fibers, yarns, color, wet processing and garments. In addition, ANN has shown its potential as a successful tool for the prediction of different textiles, fiber reinforced composites and in the evaluation of structural properties of polymer composites. The most common ANN type used in textile industry is multilayer perceptron that represents a class of feedforward ANN. A feedforward network consists of single hidden layer and sigmoid activation function is used extensively to solve textile processing problems. However, the limitations of ANN are: it is not applicable outside the data range for which it is trained. In simple, the durability of ANN is limited to the selected range of data. In addition, ANN cannot answer the relationship between input and output variables i.e., ANN cannot predict why the selected input variables result in a significant increase or decrease in output variables and vice versa. Textile processes parameters prediction in a hybrid situation is a complex task for ANN because of highly variable nature of natural fibers, spinning processes, functional materials and fabric end use requirements. The shortcoming of ANN is the implicit nature of ANN models. Rather than developing an explicit analytic expression i.e., linear or nonlinear, of input and output variables, ANN processes the variables in order to gain iterative knowledge and store it in the system. In such a case, ANN subsequently simulates the system and predicts the results.

Fuzzy logic has been applied in various fields of textiles including the prediction of melt-spun yarn count and tensile strength of fibers, classification of colored cotton into different classes, automatic recognition of fabric weave pattern and intelligent diagnosis system for fabric inspection. However, the use of fuzzy logic in a highly complex system may become an obstacle to the verification of system reliability. In addition, validation and verification of a fuzzy knowledge-based system need extensive testing with hardware. Genetic algorithms are widely used to solve various problems in textile processing right from fiber production to garment design and manufacturing. However, the major limitations of GA are: it cannot guarantee to find an optimal solution and it is time consuming. In addition, the solution quality deteriorates with the increase of the dimension of the problem. The neuro-fuzzy hybrid model was applied in several cases for the prediction of fiber, yarn and fabric properties. The prediction reliability of this hybrid model had outperformed the conventional multiple regression model and the ANN model. Supervised learning techniques such as SVM, SVR, DT, k-nearst, KNN and RT were used for classification, identification and prediction properties. However, the training for these algorithms requires a lot of computation time. Recently, deep learning has been used in some textile applications and has shown its performance in identification, defect detection and prediction. Like every method, DL has some limitations. It requires large training data to provide better performance than other methods. However, it has high computational cost to train complex data models.

Kalman filter, ensemble Kalman filter, extended Kalman filter were used to estimate and track the state of materials and their properties in fiber reinforced polymer composites. Kalman filter provides optimal solution only when the state is linear with Gaussian model. For nonlinear and non Gaussian state-space models, optimal estimation problems do not typically admit analytic solutions. Therefore, a numerical method is needed to approximate the state as EKF, EnKF and PFs. EKF relies on the linearization of nonlinear state and observations, and this may result in an erroneous estimation of the state, and in a highly nonlinear case, the filter may diverge. EnKF works better with the Gaussian model, and the accuracy of its estimation depends on number of samples. These algorithms are not effective when the model is highly non-Gaussian and/or nonlinear. The particle filters (PFs), also called Sequential Monte Carlo (SMC), are able to proceed better in these situations. PFs is a sequential Monte Carlo method to estimate the posterior density of the state in a sequential manner, and does not make any assumptions about the linearity of the system model [118].

## 4. Future Challenges

The main reason of machine learning algorithms shortcoming is to not fully utilization of valuable and persistent information about the dynamics and physiology of system. In majority of cases, the model structure of textile composites is related to physical information that may not incorporated explicitly into machine learning algorithms. Physiologically stable models, in contrast, for textiles and composites carry all available information related to system and underlying properties, can be developed using state-space framework. In general, state-space hidden Markov models allow extremely flexible frameworks for simulation and modeling of discrete time data. In a linear system with additive Gaussian noise, an optimal estimation is provided using the Kalman filter [119]. However, the textile application state-space models are highly nonlinear and may be non-Gaussian.

**Figure 4:**
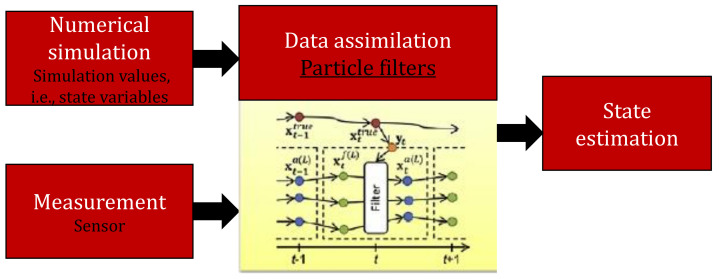
A general schematic layout of the state estimation method with data assimilation.

Sequential Monte Carlo (SMC) is a famous and reliable class of numerical methods to evaluate optimal designs related problems in nonlinear non-Gaussian systems [118]. SMC is a powerful sampling tool that works with a set of random weighted samples in order to predict the optimal solution. These samples are technically known as *particles* that are utilized during the approximation of state density and statistics of interest [118]. Given enough particles, the SMC will always perform better than the EKF or EnKF, albeit at the expense of computational requirements [119,120]. In addition, it provides the SMC converges almost to the optimal solution [121]. Figure 4 illustrates a general schematic layout of state estimation with data assimilation using SMC.

### 4.1. Classical Sequential Monte Carlo

We consider the following constrained discrete state-space model, where model representation consists of a dynamical process that captures temporal evolution of system state. As a result, the measurement model explains the relationship between the system state and the system output.

(1) $$x_{k+1}=f_{k}\left(x_{k}\right)+u_{k},$$

(2) $$y_{k}=h_{k}\left(x_{k}\right)+v_{k},$$

where $x_{k}$ is the state transition vector and $y_{k}$ is the measurement vector. $f_{k}$ and $h_{k}$ are possibly nonlinear state transition and measurement functions, respectively. $u_{k}$ and $v_{k}$ are the process and measurement zero-mean white noise sequences with known probability density functions (pdfs) $Q_{k}$ and $R_{k}$, respectively.

In the Bayesian framework, the optimal inference of the state $x_{k}$ using the measurement history $y_{1:k}=\left[y_{1},\ldots ,y_{k}\right]$ relies on the posterior density $p(x_{k}|y_{1:k})$. Using Bayes’ rule, the posterior density can be computed recursively from the following prediction and update steps:

(3) $$p\left(x_{k}|y_{1:k-1}\right)=\int_{}^{}p\left(x_{k-1}|y_{1:k-1}\right)\;p\left(x_{k}|x_{k-1}\right)\;dx_{k-1},$$

(4) $$\begin{array}{c} p\left(x_{k}|y_{1:k}\right)=\frac{p\left(y_{k}|x_{k}\right)\;p\left(x_{k}|y_{1:k-1}\right)}{\int_{}^{}p\left(y_{k}|x_{k}\right)\;p\left(x_{k}|y_{1:k-1}\right)\;dx_{k}}. \end{array}$$

In fact, the Equations (3) and (4) represent only a conceptual solution in the nonlinear case, because the integrals defined are intractable.

Sequential Monte Carlo approximate the posterior density of the unknown state using a set of *N* particles and their associated weights ${\left\{x_{k}^{\left(i\right)},w_{k}^{\left(i\right)}\right\}}_{i=1}^{N}$:

(5) $$p^{N}\left(x_{k}|y_{1:k}\right)=\sum_{i=1}^{N}w_{k}^{\left(i\right)}\delta \left(x_{k}-x_{k}^{\left(i\right)}\right),$$

where δ represents the dirac delta function. In the ideal case, the particles required to be generated from the true posterior $p(x_{k}|y_{1:k})$, which is unknown. Thereby, another distribution named *proposal distribution*$q(x_{k}|x_{k-1},y_{k})$ is used to generate the particles [118]. The importance weight of each particle $x_{k}^{\left(i\right)}$ is computed using:

(6) $$\begin{array}{c} {\tilde{w}}_{k}^{\left(i\right)}=w_{k-1}^{\left(i\right)}\frac{p(y_{k}|x_{k}^{\left(i\right)})p(x_{k}^{\left(i\right)}|x_{k-1}^{\left(i\right)})}{q(x_{k}^{\left(i\right)}|x_{k-1}^{\left(i\right)},y_{k})}, \end{array}$$

where the normalized weights are given by $w_{k}^{\left(i\right)}={\tilde{w}}_{k}^{\left(i\right)}/\sum_{j=1}^{N}w_{k}^{\left(j\right)}$.

The conditional mean estimate of the state is then given by:

(7) $${\hat{x}}_{k}=E[x_{k}|y_{1:k}]\approx \sum_{i=1}^{N}w_{k}^{\left(i\right)}x_{k}^{\left(i\right)}.$$

The weights of the particles may perish and thus require resampling [118]. The particles are resampled according to their weights, i.e., removing particles with very small weights and duplicating particles with large weights. Thus, equal weights ($\frac{1}{N}$) are assigned to all selected *N* particles. The detailed steps of sequential Monte Carlo are presented in Algorithm 1.
**Algorithm 1** Classical sequential Monte Carlo**Initialization****for** i = 1, 2, …, N **do** Generate $x_{0}^{\left(i\right)}∼N\left(x_{0}^{\left(j\right)},R_{k}\right)$. Compute the initial weights using Equation (6) and normalize.**end for****Estimation****for** k = 1, 2, …, T **do** **for** i = 1, 2, …, N **do**  Generate sample $x_{k}^{\left(i\right)}$ from the system dynamics model (1).  Compute weight using: ${\tilde{w}}_{k}^{\left(i\right)}={\tilde{w}}_{k-1}^{\left(i\right)}p\left(y_{k}|x_{k}^{\left(i\right)}\right)$. **end for** Normalize particle weights $w_{k}^{\left(i\right)}={\tilde{w}}_{k}^{\left(i\right)}/\sum_{i=1}^{N}{\tilde{w}}_{k}^{\left(i\right)}$. Resample ${\left\{x_{k}^{\left(i\right)},\frac{1}{N}\right\}}_{i=1}^{N}$. Compute the weighted mean ${\hat{x}}_{k}=\sum_{i=1}^{N}\frac{1}{N}x_{k}^{\left(i\right)}$.**end for**

### 4.2. Constrained Sequential Monte Carlo

Due to physical laws, kinematic constraints, mathematical properties such as target speed restrictions and road networks, technological limitations, geometric considerations, material balance, bounds on actuators and plants and maximum transmission capacity, various dynamical systems are limited within restricted regions [122,123,124]. Generally, these constraints may not indulged in state-space model without a major increase to avoid model complexities [119,124,125,126]. Nevertheless, it is not straightforward to take into account the physiological and modeling constraints on the state with SMC, due to the complex nature of computations in SMC. The current trend in constrained sequential Monte Carlo simply imposes the constraints on all particles of the SMC. These approaches are: (1) The acceptance/rejection approach, which enforces the constraints by simply rejecting the particles violating them [127,128]; (2) Constrained importance distribution, which imposes the constraints on all particles or equivalently sample from a constrained importance distribution [129,130,131,132,133]. The issue of how to impose the constraints -onto prior particles (particles before resampling), posterior particles (particles after resampling) or the estimated unconstrained conditional mean estimate- remains still open [129,134]. But these approach underlies the fundamental assumption that constraints on the conditional mean estimate (given in Equation (9)) can be effectively substituted by the same constraints on all particles. However, this is not true in general. It has been referred to these approaches as Point-wise Density Truncation (PoDeT) methods [135]. It was recently shown that such schemes result in incorrect estimate or irrelevant constraints altogether [135,136].

We consider a general constraint of the form [135,136]:

(8) $$a_{k}\leq \phi_{k}\left({\hat{x}}_{k}\right)\leq b_{k},$$

$\phi_{k}$ indicates the constraint function at time *k*. It is important to affirm that the constraint must only be satisfied by the state estimate provided by the conditional mean, defined as follows:

(9) $$a_{k}\leq \phi_{k}\left({\hat{x}}_{k}\right)=\phi_{k}\left(E[x_{k}|Y_{k}]\right)\approx \phi_{k}\left(\sum_{i=1}^{N}w_{k}^{\left(i\right)}x_{k}^{\left(i\right)}\right)\leq b_{k}.$$

Recently, Amor et al. derived the optimal bounds of PoDeT [135]. They revealed that error estimation was bounded by the area of state posterior density that had not included constraining interval. Specifically, if most of the density lies within the interval, i.e., the density is well-localized in the constraining interval, then the PoDeT estimation error will be small. However, if a high probability region lies outside of the interval, i.e., the density is not well-localized in the constraining interval, then the PoDeT estimation error will be large [135]. Therefore, Amor et al. proposed a new algorithm referred as “Inductive Mean Density Truncation” (IMeDeT), which inductively samples particles that are guaranteed to satisfy the constraint on the mean of the unknown state [137]. The details the steps of IMeDeT algorithm are presented in Algorithm 2. They evaluated the robustness of the proposed algorithm on the dynamic brain source localization problem using EEG data. In addition, Amor et al. introduced a novel constrained particle filter algorithm called as “mean density truncation” (MiND) and established its convergence properties [136,138]. MiND is based on the principle of minimal perturbation strategy such that the constrained posterior density is “close”to the unconstrained posterior density. Specifically, they imposed the constraint on the mean of the unknown state by perturbing the unconstrained posterior density using only one particle. The details the steps of MiND algorithm are introduced in Algorithm 3. To assess the performance of the proposed algorithm, they applied MiND to solve the problem of movement identification for forearm prosthetic control using the non-negative synergy activation coefficients. The proposed algorithm provided an accurate result with error rates significantly lower than the state-of-the-art in the literature.

**Figure 5:**
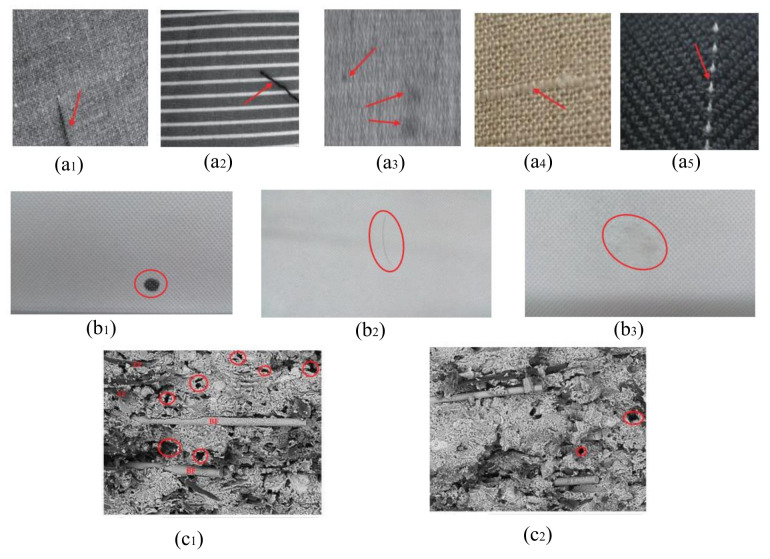
Examples of: Defective fabric samples with different patterned textures (from **a1**–**a5**). Different types of defects in cotton fabric (from **b1**–**b3**). Defect with polymer composite (**c1**,**c2**).

**Figure 6:**
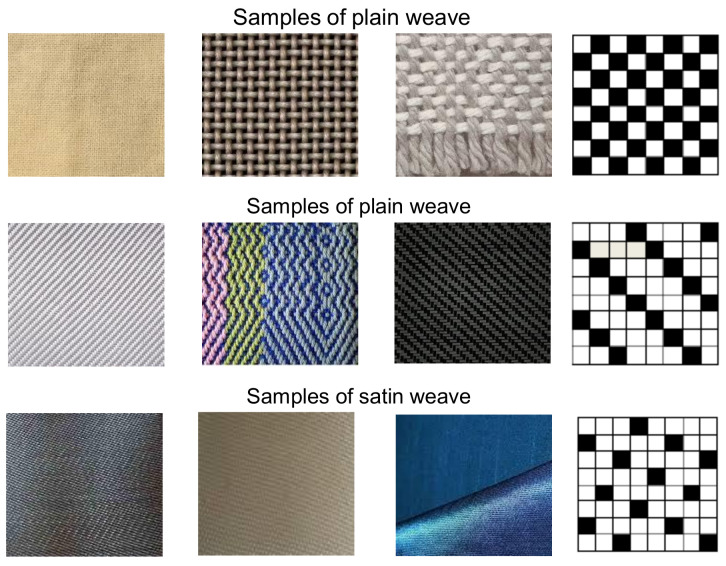
Example of identification and classification fabrics weave samples based on patterns.

Many real-world applications in textile engineering and polymer composites [139,140,141], may take benefits from this research, i.e., constrained state estimation for nonlinear and non-Gaussian dynamical systems. The main objective of this paper is to emphasize the use of sequential Monte Carlo methods as well as their constrained formulation (IMeDeT and MiND) for the development and modelling of many applications in textile engineering based on, prediction, estimation, controlling, defect detection (See examples in Figure 5), identification and classification (See example in Figure 6) etc.
**Algorithm 2** Inductive Mean Density Truncation (IMeDeT)**Initialization**Denote by $C_{k}$ the constraint region::$C_{k}=\left\{x_{k}:a_{k}\leq {\hat{x}}_{k}\leq b_{k}\right\}$.**for** j = 1, 2, …, N **do** Generate $x_{0}^{\left(j\right)}∼N\left(x_{0}^{\left(j\right)},R_{k}\right)$. Compute the initial weights using Equation (6) and normalize.**end for****Unconstrained estimation****for** k=1,2,…,T**do** **for**  j=1,2,…,N **do**  Generate sample $x_{k}^{\left(i\right)}$ from the system dynamics model (1).  Calculate the weights $w_{k}^{\left(j\right)}$ of $x_{k}^{\left(j\right)}$ using Equation (6); then, normalize the weights.  
**Constrained estimation**  **for** i = 1, 2, …, j **do**   **if** $\sum_{i=1}^{j}w_{k}^{\left(i\right)}x_{k}^{\left(i\right)}\in C_{k}$ **then**    Go to the next step.   **else**    Find a particle $x_{k}^{\left(j\right)}$ such that $\sum_{i=1}^{N}w_{k}^{\left(i\right)}x_{k}^{\left(i\right)}\in C_{k}$.   **end if**  **end for** **end for** Compute the constrained weighted mean ${\hat{x}}_{k}=\sum_{i=1}^{N}w_{k}^{\left(i\right)}x_{k}^{\left(i\right)}$.**end for**

**Algorithm 3** Mean Density Truncation (MiND)

**Initialization**

The same as initializing IMeDeT.
**for** k = 1, 2, …, T **do**
 **Unconstrained estimation**
 **for** j = 1, 2, …, N **do**
  Generate sample $x_{k}^{\left(j\right)}$ from the system dynamics model (1).
  Compute weight using Equation (6).
 **end for**
 Normalize particle weights $w_{k}^{\left(i\right)}={\tilde{w}}_{k}^{\left(i\right)}/\sum_{j=1}^{N}{\tilde{w}}_{k}^{\left(j\right)}$.
 Resample ${\left\{x_{k}^{\left(i\right)},\frac{1}{N}\right\}}_{i=1}^{N}$.
 Compute the weighted mean ${\hat{x}}_{k}=\sum_{i=1}^{N}\frac{1}{N}x_{k}^{\left(i\right)}$.
 
**Constrained estimation**
 **if** ${\hat{x}}_{k}\;\notin C_{k}$ **then**
  Remove the furthest particle $x_{k}^{\left(i\right)}$.
  Add a new particle $x_{k}^{N}$ using $a_{k}\leq \frac{1}{N}\sum_{i=1}^{N-1}x_{k}^{\left(i\right)}+\frac{1}{N}x_{k}^{\left(N\right)}\leq b_{k}$ and $a_{k}^{\prime }\leq x_{k}^{\left(N\right)}\leq b_{k}^{\prime }$ where $a_{k}^{\prime }=Na_{k}-\sum_{i=1}^{N-1}x_{k}^{\left(i\right)}$ and $b_{k}^{\prime }=Nb_{k}-\sum_{i=1}^{N-1}x_{k}^{\left(i\right)}$.
  Compute the constrained weighted mean ${\hat{x}}_{k}=\sum_{i=1}^{N}\frac{1}{N}x_{k}^{\left(i\right)}$.
 **end if**

**end for**



## 5. Future Direction and Summary

This study focuses on machine learning classification methods specifically designed for textiles and polymer composites. It elucidates how classification methods are applied in fiber reinforced composites to deal with problems. Based on discussed literature, this study clearly explains that machine learning classification receives significant consideration in textiles and composites industries. SVM and ANN are widely used classification methods as they provide better prediction accuracy. In addition, this study provides the classification of carbon fiber reinforced composites and the inclusion of polymeric fibers in composites formation. It elaborates recently used advanced machine learning algorithms for textile processes and carbon fiber reinforced composites. It provides critical and in-depth information regarding the algorithms applied during yarn production, fabric manufacturing and textile finishing processes. Drawbacks and limitations of each method are discussed in detail. This study proposes gateway and opens new avenues not only for researcher community but also for the readership of the journal. In addition, we suggest the use of sequential Monte Carlo methods, i.e., particle filters for control, monitoring, prediction, and identification, in textiles and composites.

For future work, some novel algorithms e.g., golden eagle optimiser are suggested to study the performance of fiber reinforced polymer composites, besides the classification method. Golden eagle optimiser is a recent method and only one or two studies have applied it so far. This can be beneficial to discover complex relationships and useful patterns between textiles and fiber reinforced polymer composites.

In textiles and composites industries, researchers mostly used single classifier. However, combining multiple algorithms may provide a more accurate and semantic vision for the classification of textile processes. Therefore, researchers should start to use hybrid models in order to achieve better results.

## Figures and Tables

**Table 1:** A comparison of previously performed work for defects detection in textile processes.

Proposed Models	Purposes	Methods	Major Findings	Authors
Gabor filters and pulse coupled neural network (PCNN)	Fabric defect detection for of warp knitting fabrics	Enhanced the image contrast using Gabor filters and they applied PCNN for segmentation purpose	Results of the experiments have demonstrated that the proposed PCNN with Gabor has higher detection accuracy (98.6%)	Li et al. [81]
Convolutional neural networks (CNN)	Automatic quality control for fiber placement manufacturing	A pixel-by-pixel classification has been created for the defects of the whole part scan	Simulation results showed that the proposed strategy failed to achieve satisfactory results due to their small training dataset (confront with over-fit problem)	Sacco et al. [82]
Fuzzy ARTMAP neural network	Evaluation of yarn surface qualities based on the extracted features	Wavelet texture analysis, attention-driven fault detection, and statistical measurement are used to extract the characteristic features of yarn surface appearance from images. and a fuzzy ARTMAP neural network is employed to classify and grade yarn surface qualities based on the extracted features.	The experimental results showed that the fuzzy ARTMAP achieved superior results to classify yarn surfaces compared to ANN and SVM	Liang et al. [83]
CNN	Fabric defect detection	Mobile-Unet is used to improve the performance of CNN	Experimental results showed that the detection speed and the segmentation accuracy in the proposed method achieve powerful performance compared to SegNet and U-net	Jing et al. [84]
CNN	Fabric defect detection and classification system	(1) Prototyped an advanced image acquiring model using National Instruments NI Vision; (2) Train the CNN using standard textile fabrics. (3) Testing fabrics are examined by the trained CNN.	The experiment work produced good accuracy in defect detection compared to the Bayesian classifier and SVM methods. In addition, it provided better processing and classification on defective pattern variation in patterned fabric	Jeyaraj et al. [85]
CNN	Fabric texture defects classification	Compressive sampling theorem is used to compress and augment the data in small sample sizes	The classification results of the proposed model achieved higher accuracy 97.9% compared to KNN, ANN and SVM	Wei et al. [86]
Deep convolutional generative adversarial network	Localize the surface defects for woven fabrics	A new encoder block was used to reconstruct query image with normal texture and no defect	The experiments results showed that the proposed approach is not sensitive to image blurring or illumination changes. In addition, it has high flexibility and high detection accuracy for different types of texture structures and defects.	Hu et al. [87]

**Table 2:** Comparative study of related work in fiber reinforced polymer composites.

Proposed Models	Purposes	Methods	Major Findings	Authors
Deep neural network (DNN) with finite-element method	Estimation of the stress distributions of the aorta	DNN model was constructed and trained, where the input is the results obtained by finite-element analysis method and the output was the aortic wall stress distributions	Simulations results showed that the proposed model was able to predict the stress distributions with lower error and accurate surrogate of finite-element analysis for stress analysis	Liang et al. [105]
Logical analysis of data (LAD)	Process control technique applied to the routing process for CFRP	Monitoring and evaluating the quality of the machined parts in CFRP by controlling some machining features and parameters	Experimental work showed that the proposed LAD outperformed ANN in both accuracy of controlling and monitoring variables	Shaban et al. [106]
Neural network regression	Damage location detection of the CFRP composite plate	Process the signals obtained by acoustic emission sensors in CFRP composite	Experiments are applied on the composite structure showed that the proposed approach provided a good result in the estimation of localization of damage signals comparing to the actual sources	Zhao et al. [107]
ANN	Prediction of damage progression and fatigue life in laser induced graphene interlayered fiberglass composites	Investigation of the potential of exploiting the piezoresistive properties of laser induced graphene interlayered fiberglass composites	Simulation results showed that piezoresistive laser induced graphene interlayers provided high prediction accuracy of fatigue life in multifunctional composite structures	Nasser et al. [108]
ANN, SVM and extreme learning machine	Assessment of delamination damage in fiber-reinforced polymer composite beams	Machine learning algorithms have been adopted as inverse algorithms to evaluate the delamination parameters	Experimental results demonstrated that the SVM provided the best prediction accuracy compared to ANN and extreme learning machine algorithms for delamination damage in fiber-reinforced polymer composites	He et al. [97]
Generative kernel principal component thermography and spectral normalized generative adversarial network	Defect detection in carbon fiber reinforced polymer composites	Extraction of nonlinear features from thermographic data and producing a number of informative thermographic data to improve the defect detection	Testing results showed that the proposed approach improved the detection accuracy of subsurface defects in CFRP	Liu et al. [109]
Bayesian regularized neural network	Weld quality classification for ultrasonic welding of CFRP	Proposing a feature selection methodology that combines new clustering overlap analysis with Fisher’s ratio to improve the classification results	Simulations results in this application showed that the Bayesian regularized neural network have higher robustness and classification accuracy compared to SVM and kNN	Sun et al. [110]

**Table 3:** Techniques used in textiles and fiber reinforced polymer composites.

Classes	Techniques	Applications
Yarn manufacturing	ANN, FL, GA and SVM	Prediction properties
Fabric manufacturing	DL, ANN, FL, DE, GA, SVM, DT, K-nearst, KNN, RF and SVR	Prediction, classification and recognition, optimization, identification and estimation
Finishing processes	ANN and image processing analysis	Prediction, defect detection
Textile based composites	ANN, DL, KF, EKF, EnKF, FEA, DT, KNN, RT and SVR	Prediction, defect detection, control, classification, estimation, tracking

## Data Availability

Not applicable.
